# Intraoperative central venous pressure during cardiopulmonary bypass is an alternative indicator for early prediction of acute kidney injury in adult cardiac surgery

**DOI:** 10.1186/s13019-024-02734-7

**Published:** 2024-04-23

**Authors:** Lei Wang, Lanxin Hu, Qiong yan Dai, HaoYu Qi, ZhenHong Wang, Xin Chen

**Affiliations:** 1 Department of Thoracic and Cardiovascular Surgery, Nanjing First Hospital, Nanjing Medical University, Chang le Road 68, Nanjing, Jiangsu China; 2 Department of Anesthesiology, Nanjing First Hospital, Nanjing Medical university, Nanjing, Jiangsu China; 3 Department of Anesthesia, Jiangning Hospital Affiliated to Nanjing Medical College, Nanjing, Jiangsu China

**Keywords:** Acute kidney injury, Central venous pressure, Cardiac surgery, Venous congestion, Cardiopulmonary bypass

## Abstract

**Background:**

The relationship between venous congestion in cardiopulmonary bypass (CPB) and acute kidney injury (AKI) in cardiac surgery has not utterly substantiated. This study aimed at investigate the relationship between CVP in CPB and the occurrence of AKI.

**Methods:**

We retrospectively reviewed 2048 consecutive patients with cardiovascular disease undergoing cardiac procedure with CPB from January 2018 to December 2022. We used the median CVP value obtained during CPB for our analysis and patients were grouped according to this parameter. The primary outcomes were AKI and renal replacement therapy(RRT). Multivariable logistic regression was used to explore the association between CVP and AKI.

**Results:**

A total of 2048 patients were enrolled in our study and divided into high CVP group (CVP ≥ 6.5 mmHg) and low CVP group (CVP < 6.5 mmHg) according to the median CVP value. Patients in high CVP group had the high AKI and RRT rate when compared to the low CVPgroup[(367/912,40.24%)vs.(408/1136,35.92%),*P* = 0.045;(16/912,1.75%vs.9/1136;0.79%), *P* = 0.049]. Multivariate logistic regression analysis displayed CVP played an indispensable part in development of renal failure in surgical.

**Conclusions:**

Elevated CVP(≥ 6.5mmH_2_OmmHg) in CPB during cardiac operation is associated with an increased risk of AKI in cardiovascular surgery patients. Clinical attention should be paid to the potential role of CVP in predicting the occurrence of AKI.

As technologies in medicine have advanced, negative cardiac surgery complications, such as mortality within hospitals, have reduced significantly [1, 2]. Nevertheless, acute kidney injury (AKI) continues to be the most prevalent and severe complication. A recent survey found that the incidence of cardiac surgery-associated AKI(CSA-AKI) fluctuates from 10 to 40% according to various definitions of AKI [3, 4]. Potential CSA-AKI procedures are associated with diminished perfusion during CPB. Actual studies have verified that renal venous congestion is essential for CSA-associated negative kidney events’ progression, while a retrospective study found that increased central vein pressure (CVP), as opposed to arterial blood pressure or cardiac index, is related to reduced GFR in cardiovascular patients undergoing surgery [5, 6].

According to the logic underlying the association between CVP and AKI among patients who are critically ill, a high CVP can impede renal venous return to the reservoir and disrupt microcirculatory flow of blood, thereby impairing organ function and increasing the risk of death. Moreover, elevated CVP indicates inadequate venous drainage in CPB will affect renal venous blood drainage and lead to renal congestion, resulting in deterioration of acute kidney injury (AKI) [7]. Nonetheless, unknown is the correlation between CVP in AKI and CPB among cardiac patients.

Unfortunately, studies regarding the relationship between CVP and renal function protectable in CPB are originated from post-CPB so that miss the relevant research during CPB. Accordingly, we conducted this study to investigate the relationship between high CVP during AKI and CPB among cardiac patients upon adulthood, with the hypothesis that CSA-AKI in these patients is related to venous obstruction during CPB.

## Patients and methods

The present study was approved by the Regional Human Research Ethics Committee of Nanjing First Hospital. Reference number: KY20220805-03.

### Definition of CVP in CPB

The CVP value was conducted using electronic health records (EHRs). After calculation, the median CVP value of 2048 patients was 6.5 mmH_2_O. Therefore, CVP ≥ 6.5mmH_2_O was defined as high CVP (*n* = 912) and CVP < 6.5mmH2O as low CVP(*n* = 1136).

### Inclusion and exclusion criteria

The following were the criteria for inclusion: (1) patients at least 18 years; (2) patients who did not undergo the incision surgery at the right atrium, such as isolated CABG(coronary artery bypass grafting), isolated AVR(aortic valve replacement), aortic-related surgery(aortic ascending replacement, Wheat procedure, Bentall procedure) and AVR + CABG;3) Complete the entire treatment process in our hospital; Patients who met the following criteria were excluded: 1) individuals younger than 18 years old;2) discharged or died within 48 h after operation, (3) Preoperative right heart failure;4) Preoperative renal insufficiency[those whose preoperative creatinine value(133 > µmol/L) exceeded the normal range or had a history of nephrotic syndrome.]; 5) Preoperative right ventricular area change rate is less than 35%. This study was approved by the Institutional Review Board at Nanjing Medical University in compliance with Health Insurance Portability and Accountability Act regulations and the Declaration of Helsinki.

### Surgical technique

During the operation, intravenous inhalation and anesthesia were used routinely. Blood pressure in the left radial artery and oxygen saturation in the veins of peripheral tissues is measured. In the procedure, esophageal ultrasound and the bispectral index were utilized to assess postoperative cardiac function and intraoperative anesthesia depth. The bladder was implanted with a foley catheter equipped with a temperature probe to monitor the patient’s central body temperature. In our center, the right internal jugular vein is routinely selected as the CVP measurement channel, and CVP measurement is performed after the venous catheter is placed. We continuously recorded the CVP value during CPB and obtained the average value.

## CPB management

### Artery cannula and venous cannulas

In numerous cases, the aortic artery cannula is the primary choice for perfusion. The femoral artery was selected as the intraoperative perfusion vessel in some ascending aorta cases. A 2-stage venous cannula (28/32# for ≤ 80 kg, 32/40# for>80 kg; Wei Gao, Shan Dong, China) were implemented to manage venous drainage during CPB. The patient’s BMI was considered when selecting a cannula size.

Venous drainage and CPB related parameters.

CPB was initiated by draining the venous blood of the patient into a venous reservoir (max volume: 4.5 L) and completing oxygenation with a hollow fiber membrane oxygenator (Terumo, Japan). The intraoperative level of the venous reservoir is maintained at least 1000 ml, and if the venous volume is less than 1 L, we will use VAVD(vacuum-assisted venous drainage) to assist drainage. During CPB, the VAVD (Medtronic, America) approach a negative pressure target between − 30 mmHg and − 60 mmHg were utilized. A Stockert S5 roller pump was used to transport arterial blood to the vascular system, and a heat exchanger was used to maintain a constant body temperature. Priming extracorporeal circuit was realized with Plasma-Lyte A (500 mL), Hydroxyethyl starch 130/0.4 (1 L), a hematocrit of at least 20% with red blood cells, plasma products, 20% albumin, mannitol, and heparin with low molecular weight. CPB was managed to use goal-directed perfusion (IDO_2_ ≥ 280 mL/minute/m^2^, IDO_2_: oxygen delivery index). Using an alpha-stat strategy, the blood gases were kept between 35 and 45 mmHg of PCO_2_. Upon CPB, an average perfusion pressure of 40 to 60 mmHg must be maintained.

### Primary outcomes

Acute kidney dysfunction, such as AKD and AKI, was the primary result. AKI was defined using the KDIGO (Kidney Disease, Improving Global Outcomes) standard. First, within 48 h of surgery, the creatinine level rose by at least 0.3 mg/dl or at least 26.5 µmol/L. Second, a known or suspected rise in creatinine to at least 1.5 times the baseline within the prior week. Third, a urine volume of less than 0.5 ml/kg/h for more than 6 h [8]. Moreover, the average central venous pressure and the RRT requirement were assessed.

### Statistical analysis

For data analysis, SPSS Statistics version 24.0 (SPSS, Chicago, IL, USA) was utilized. The patient’s attributes were expressed using the median and interquartile range (IQR). Non-parametric analyses analyzed the distinctions between the two groups and the associations between various indices. The incidence of variables in contingency tables was evaluated by means of Fisher’s exact test or the χ2 test. Utilizing univariate analysis screening, variables with substantial differences and prior studies’ suggestions were selected. After the indicators associated with AKI incidence became part of the Logistic multivariate regression analysis, the risk factors and respective coefficients were determined, and the prediction model for AKI was developed. The risk association between CVP during AKI and CPB was assessed using multiple logistic regression. In all analyses, *P*<0.05 was deemed to have statistical significance.

## Results

### Acute adverse kidney events and RRT

**Fig. 1:**
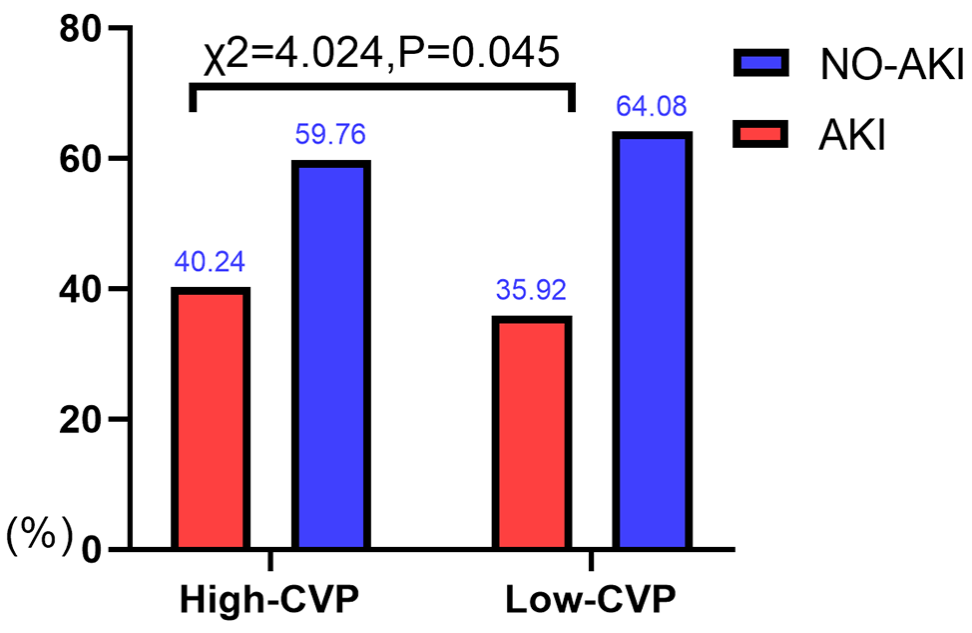
Incidence rates of AKI in two groups

**Fig. 2:**
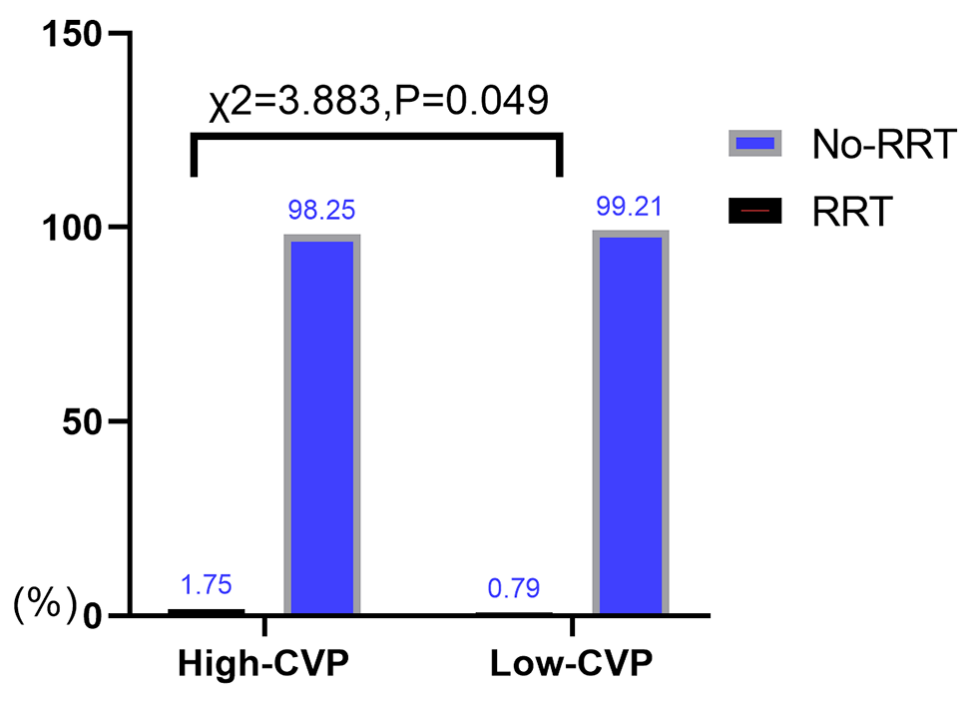
Incidence rates of RRT in two groups

**Fig. 3:**
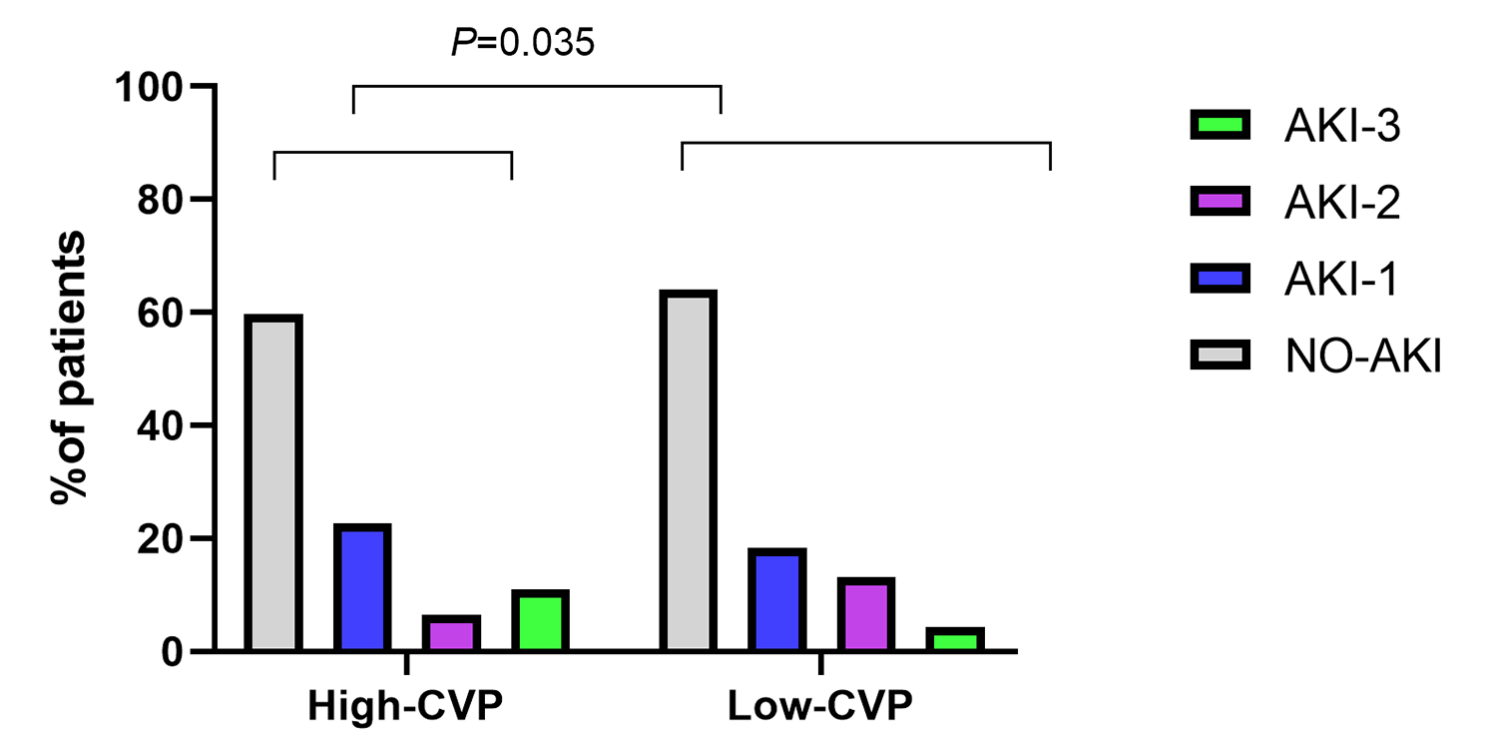
Stage of AKI in two group

775 out of 2,048 (37.84%) patients were verified to have AKI, and 25 patients acquired renal replacement therapy (3.23%). Incidence of AKI and RRT(renal replacement therapy, RRT) acquired in High-CVP group were higher than that in Low-CVP group [(367/912,40.24%) vs. (408/1136,35.92%), *P* = 0.045; (16/912,1.75% vs. 9/1136;0.79%), *P* = 0.049, respectively]. Moreover, patients in the high CVP group experienced more severe of AKI compared to those in low CVP group (*p* = 0.035; Figs. 1, 2 and 3).

### Baseline characteristics

As provided in Table 1, there were substantial variations between the two groups’ baseline attributes. Patients with elevated CVP tended to be geriatric male. On admission, their BUN, BMI, cardiopulmonary bypass duration, the hypertension history, intraoperative blood product infusion, postoperative mechanical ventilation duration, and vacuum-assisted venous drainage (VAVD) was significantly higher.

**Table 1:** Characteristics of the cohort

Characteristics	High-CVP group (*n* = 912)	Low-CVP group (*n* = 1136)	*P* value
Age(>65years)	375	404	0.010
Male, (n) %	479(61.81)	267(20.97)	<0.001
BMI(kg/m2), median(IQR)	28.4(2.7)	25.0(2.9)	0.037
Hypertension(n) %	389(50.19)	239(18.77)	0.000
Diabetes, (n) %	20(2.58)	43(3.37)	0.491
Creatinine(mg/dl), median(IQR)	0.84(0.6)	0.85(0.5)	0.842
BUN(mg/dl), median(IQR)	85(24)	84(25)	0.757
eGFR(ml/min/1.73m2), median(IQR)	114(4)	115(5)	0.738
CPB time(min), median(IQR)	117(5)	88(6)	0.001
MAP during CPB(mmHg), median(IQR)	53(2)	52(3)	0.484
VAVD used(n) %	39(4.27)	100(8.80)	0.000
Aortic cross-clamp time(min), median(IQR)	65(13)	66(14)	0.356
HCT(%),median(IQR)	23(2)	24(3)	0.568
RBCs transfused(n) %	88(11.75)	92(7.22)	0.000
COPD(n)	23	49	0.377
High cholesterol	24	32	0.798
EF(%),median(IQR)	53(5)	54(6)	0.457
Intraoperative fluid input volume(ml), median(IQR)	2160(187)	2165(188)	0.663

EF: ejection fraction; BMI: Body mass index; BUN: Blood urea nitrogen; eGFR: estimated Glomerular filtration rate; CPB: Cardiopulmonary bypass; MAP: Mean average pressure; CVP: Central venous pressure; VAVD: Vacuum-assisted venous drainage; HCT: Hematocrit; RBCs: Red blood cells; COPD: chronic obstructive pulmonary disease; IQR: Interquartile range

### Operative data and postoperative variables

The aggregate operative mortality rate was 3.81% (78/2048). No substantial difference existed among the groups regarding operative mortality. Mortality in AKI (40/775) group is higher than that in NO-AKI group [(40/775,5.16%) vs. (38/1237,3.07%), *P* = 0.012]. 44 patients dead in High-CVP group and 34 patients in Low-CVP group (infections, cardiogenic shock, low cardiac output syndrome, multi-organ failure, etc.).Operation classification and perfusion data are displayed in Table 2. There is no significant difference when it comes to MAP, urinary output, CPB time, aortic cross-clamp time, rate of defibrillations, and surgical classification between two groups(all *P*>0.05).

**Table 2:** Operative data

Characteristics	High-CVP group *n* = 912	Low-CVP group *n* = 1136	*P* value
Surgical classification			
Isolated CABG surgery, n (%)	406(44.52)	562(49.48)	0.849
Isolated aortic valve surgery, n (%)	305(33.44)	401(35.30)	0.903
CABG and aortic valve surgery, n (%) Vascular-related surgery, n(%)	100(10.97) 101(11.07)	72(6.35) 78(6.87)	0.952 0.887
MAP during CPB, median(IQR)	54(6)	55(6)	0.366
Mean pump flow(L/min.m2), median(IQR)	2.5(0.2)	2.5(0.3)	0.676
HCT(%),median(IQR)	23(4)	24(3)	0.443
RBCs transfused(n,%)	18(1.97)	29(2.55)	0.370
Defibrillation (n)	92(10.09)	123(10.83)	0.258
Urinary volume(ml), median(IQR)	818(77)	820(75)	0.348
Furosemide prescribed(n,%)	48(5.26)	66(5.81)	0.089
Low output syndrome(n,%)	3(0.33)	8(0.70)	0.445
Cerebrovascular accidents(n) Temporary(POCD/TIA)	56/3	72/7	0.773
Permanent(cerebral hemorrhage/stroke)	4/2	6/3	0.851
Ultrafiltrate output(ml), median(IQR)	687(36)	704(42)	0.538

POCD: Postoperative cognitive dysfunction; TIA: Transient ischemic attack; HCT: Hematocrit; RBCs: Red blood cells; MAP: Mean average pressure; CABG: Coronary artery bypass grafting

**Table 3:** Multivariable analysis determining covariate factors associated with AKI development in eligible subjects

	All eligible subjects(*n* = 2048)			
Variable	standardizedβ	OR	95%CI	*P*-value
Male	0.532	1.77	1.27–2.79	0.046
BMI	0.344	1.53	1.05–3.68	0.039
Age>65 years	0.731	1.91	1.28–2.90	0.015
Hypertension	0.578	1.54	1.06–2.81	0.001
Red blood cell transfused	0.536	1.71	1.14–2.44	0.001
CPB duration	0.758	1.98	1.53–3.73	<0.001
Mechanical ventilation time	0.119	1.03	1.16–2.44	0.045
VAVD used	-4.19	0.74	0.28–0.96	0.003
High-CVP	0.771	2.01	1.56–3.54	0.017

BMI: Body mass index; CPB: Cardiopulmonary bypass; CVP: Central venous pressure; VAVD: Vacuum-assisted venous drainage

### Multivariable logistic regressions examining risk and protective factors for AKI in all subjects

**Fig. 4:**
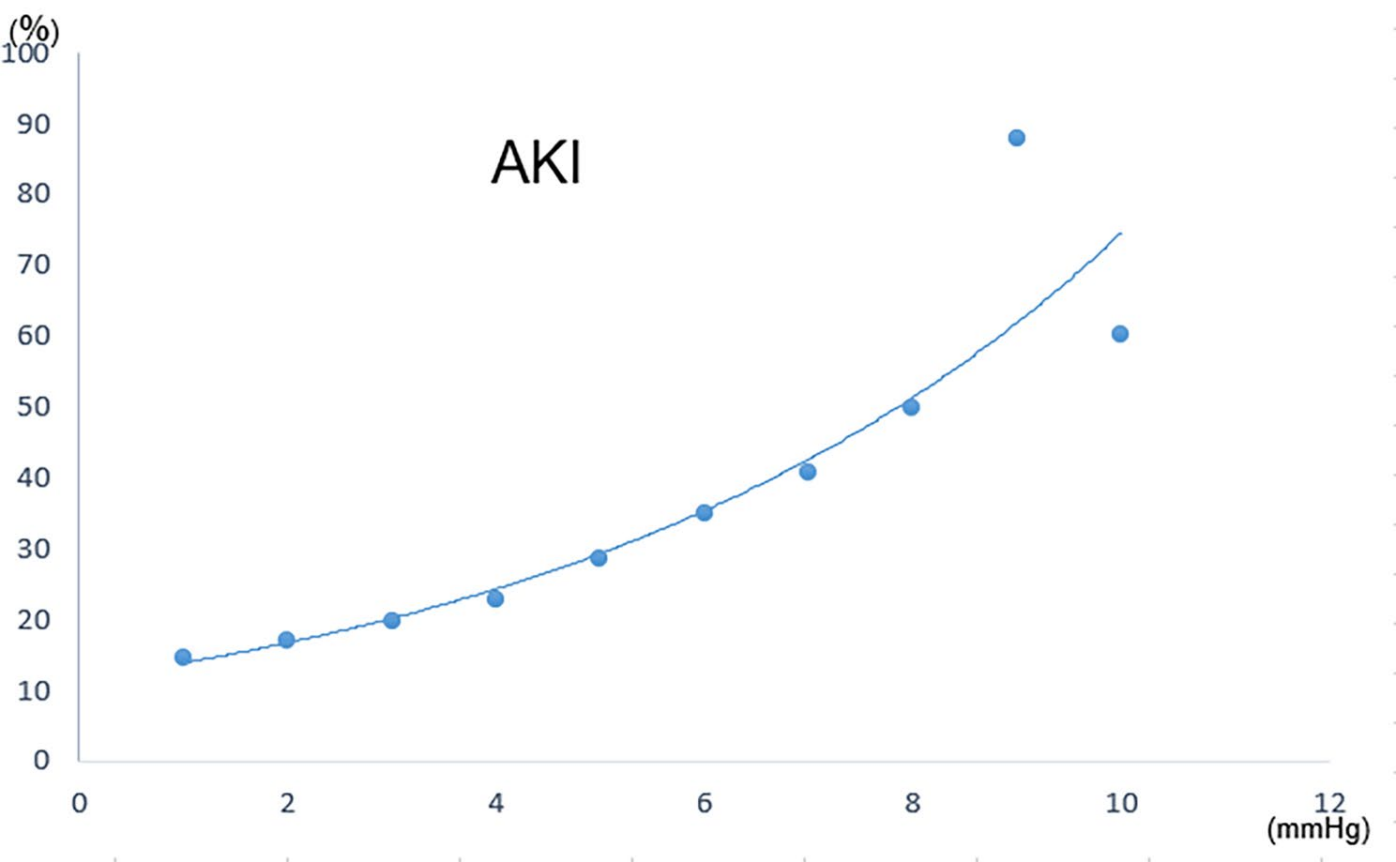
The linear relationship between CVP and AKI incidence

Using multivariate logistic regression, prospective protective and risk factors in AKI progression were identified. As demonstrated in the data below, a low CVP may be protectively against CSA-AKI. The association between CVP and AKI incidence is depicted in Fig. 4. Such incidence rose as CVP rose, particularly when CVP exceeded 5 mmHg. Meanwhile, age>65years, high BMI, hypertension, prolonged CPB, prolonged postoperative mechanical ventilation time, RBCs transfused and VAVD were independently correlated with CSA-AKI. (Table 3)

## Discussion

Approximately 30-40% of patients who underwent cardiac surgery experience AKI, and almost 2-6% of AKI will require hemodialysis [9, 10]. Based on the findings of our study, with respect to the 2012 KDIGO diagnostic AKI criteria, the incidence rates of CAS-AKI and RRT are, respectively, 37.84% and 3.23%, consistent with prior research [11, 12].

CSA-AKI pathophysiology is not completely understood, but includes nephrotoxin, hemodynamic disturbance, inflammatory response, hypoxia, CPB, and neuroendocrine activation [13, 14]. Contemporary studies affirm that renal venous congestion is essential in negative kidney events’ development associated with cardiac surgery, while a retrospective study found that increased CVP, rather than systolic blood pressure or cardiac index, is related to reduced GFR among patients subjected to cardiovascular surgery [15].

CVP (normal 5–12 mmHg) is a pressure measured from the superior vena cava or right atrium that denotes the pressure index of cardiac preload and equivalent to the right ventricle end-diastolic pressure [16]. Clinicians utilize CVP to assess venous congestion in CPB patients with cardiac disease. In fact, CVP has been criticized as an unreliable indicator of venous congestion due to a number of aspects, such as the height difference between the intravenous catheter and the barometer, the size of the venous cannula, the resistance of the pump line, and the height discrepancy between the patient’s entrance and the venous reservoir’s line [17]. CVP can be a useful assessment for venous obstruction, despite the valid criticism, when the aforementioned variables are considered. Cardiac surgery requires effective venous drainage to complete the CPB and thus affects renal venous congestion. For no-right atrium incision surgery, the residual blood in the right atrium more or less affects the venous drainage, resulting in varying degrees of congestion (CVP>5mmHg)in the renal veins so as to result in renal injured [18]. To our knowledge, our study results showed that the incidence of AKI and RRT in patients with low-CVP was lower than that of patients with high-CVP, preliminarily confirming the potential correlation between venous drainage and AKI.

These factors may have enabled this event. First, reduced blood flow in the kidney can lead to neutrophil accumulation in the peritubular capillaries [19, 20]. Second, decreased renal blood flow induced upregulation of inflammation signaling (e.g., NF-kB), thus elevating neutrophil adhesion to activated endothelial cells [21]. Moreover, investigations have demonstrated that pharmacologic inhibition of inflammation signalling ameliorates congestion-mediated kidney injury deterioration [22]. Meanwhile, our study indicated that VAVD can decrease CVP via negative pressure of venous reservoir in CPB by increasing venous drainage, thereby defending renal function during cardiac surgery [23, 24].

We continue to analyze other effects of CSA-AKI and explore potential mechanisms. Due to hemolysis, iron burden, and pro-inflammatory aspects, RBCs transfused intraoperatively can cause renal injury [25]. Patients on prolonged mechanical ventilation often develop pulmonary complications. This may alter the oxygen delivery to tissues and organs such as the kidneys. On the other hand, it can increase the frequency of pulmonary infections, require antibacterial treatment, increase burden against renal drug metabolism, and eventually result in kidney impairment [26]. Elderly patients (above 65 years) typically have diminished renal reserve capacity, are predisposed to insufficiency of other organs, and are more susceptible to factors like hypoxia and ischemia [27]. Obesity is related to AKI following cardiac surgery that can be caused by BMI. Perfusion hyperfiltration increases the risk of kidney damage; oxidative stress and inflammatory response likewise has involvement [28]. Males have a high BMI and are frequently related to androgens. Androgen can substantially increase blood vessels’ receptiveness to vasoactive substances, reduce prostacyclin levels, and enable thromboxane production. In addition, it can cause microthrombosis and elevate platelet aggregation, impairing renal blood flow and resulting in renal injury [18]. The relationship between hypertension and renal damage is based on the progressive arteriosclerosis of all renal vessels in hypertensive patients, the changes of renal hemodynamics caused by lumen stenosis and glomerular ischemia, and the activation of renin-angiotensin-aldosterone system, which leads to renal damage [29].

### Comment and limitations

It is considered that CVP is an alternative indicator for early prediction of acute kidney injury due to the linear relationship between CVP and the incidence of AKI. Some suggestions should be given to reduce the risk of AKI after cardiac surgery, such as by using a large bore venous cannula, increasing the height of the operating table and actively using ultrafiltration during or after CPB. However, objective limitations can not be neglected. Our investigation is retrospective at one point, which indubitably introduces bias. In addition, individual differences in baseline CVP values are typical yet frequently unavailable among clinical aspects, which is another design limitation. In a future prospective study, the relationship of CVP in CPB and CSA-AKI in other cardiac surgeries will be an important supplement to improve the conclusion of this study. At last, the central venous catheter was placed in the superior vena cava at a depth of about 13–15 cm, which may lead to inaccurate measurement of CVP.

## Data Availability

Not applicable.
